# Full Characterization of Thrombotic Events in All Hospitalized COVID-19 Patients in a Spanish Tertiary Hospital during the First 18 Months of the Pandemic

**DOI:** 10.3390/jcm11123443

**Published:** 2022-06-15

**Authors:** Álvaro Tamayo-Velasco, Carolina Bombín-Canal, María José Cebeira, Laura Sánchez-De Prada, José Pablo Miramontes-González, Marta Martín-Fernández, María Jesús Peñarrubia-Ponce

**Affiliations:** 1 Department of Haematology and Hemotherapy, Hospital Clínico Universitario de Valladolid, 47003 Valladolid, Spain; alvarotv1993@gmail.com (Á.T.-V.); cbombin@saludcastillayleon.es (C.B.-C.); mjcebeira@saludcastillayleon.es (M.J.C.); mpenarrubia@saludcastillayleon.es (M.J.P.-P.); 2 Centro de Investigación Biomédica en Red de Enfermedades Infecciosas (CIBERINFEC), Instituto de Salud Carlos III, 28029 Madrid, Spain; 3 BioCritic, Group for Biomedical Research in Critical Care Medicine, 47005 Valladolid, Spain; 4 Department of Microbiology, Hospital Clínico Universitario de Valladolid, 47003 Valladolid, Spain; lsanchezd@saludcastillayleon.es; 5 Instituto de Investigación Biomédica de Salamanca (IBSAL), Universidad Pontificia de Salamanca, 37002 Salamanca, Spain; jpmiramontes@hotmail.com; 6 Department of Internal Medicine, Hospital Universitario Río Hortega, 47012 Valladolid, Spain; 7 Department of Medicine, Toxicology and Dermatology, Universidad de Valladolid, 47003 Valladolid, Spain

**Keywords:** thrombotic events, COVID-19, D-dimer, thrombophilia, diagnosis, prognosis

## Abstract

The presence of a procoagulant state, COVID-19-related coagulopathy, and an increased rate of thrombotic events (TEs) is widely known about. However, descriptive studies are scarce. Here, we conducted a large retrospective study including 2894 hospitalized COVID-19 patients followed up during the first 18 months of the pandemic to completely characterize any TE. Major TEs showed a 3.45% incidence rate. TEs were associated with increased intubation/90-day mortality risk [OR = 1.71, 95% CI (1.12–2.61), *p* < 0.013]. Venous thrombotic events (VTEs) were more frequent than arterial thrombotic events (ATEs) (72% vs. 28%), associated with enhanced levels of D-dimer (cross-linked fibrin derivatives formed during thrombolysis), which were related to mortality but more useful for early detection of thrombosis. In this regard, D-dimer plasma levels above 2014 µg/mL at hospital admission identify TEs with 91% accuracy (AUC = 0.91, *p* < 0.001), rising to almost 95% (AUC = 0.94, *p* < 0.001) with a cut-off value of 2666 µg/mL in VTEs. Moreover, 41% of TEs occurred in patients receiving LMWH thromboprophylactic treatments in hospital or domiciliary therapies. SARS-CoV-2 infection along with a sedentary lifestyle derived from the confinement in 2020 could be more determinant than a procoagulant state in patients with risk factors for TEs. Furthermore, the normal results obtained from the thrombophilia study after the acute process are linked to this independent procoagulant state and to SARS-CoV-2-derived coagulopathy.

## 1. Introduction

Coronavirus disease 2019 (COVID-19) is an infectious process associated with important multisystemic clinical manifestations [1,2]. It is responsible for high rates of hospitalization, intensive care unit (ICU) admission, morbidity, and mortality [3,4,5]. Symptoms and complications are mainly related to respiratory failure; however, a wide variety of them have been described [4]. Moreover, the presence of immune dysregulation with abnormal cytokine release [6], endothelitis [7], and coagulopathy [8] has been extensively described in severe cases. Thus, these immunological alterations could enable one to categorize COVID-19 into different phenotypes [4] and to find specific diagnosis, prognosis, and therapeutic biomarkers [9].

Since the beginning of the pandemic in China, the presence of alterations in coagulation parameters has been described [8,10]. The initial phase of the infection is associated with increased levels of D-dimer and fibrinogen, whereas activated partial prothrombin time, prothrombin time, and platelet counts are often normal [11]. This coagulopathy creates a procoagulant state that impairs host defense mechanisms, immunity, and the coagulation system [11]. Thus, thrombotic events (TEs) became related to COVID-19, and the risk of them increases according to viral infection severity and ICU admission [12,13].

Therefore, protocolized treatments recommended low-molecular-weight heparin (LMWH) thromboprophylaxis in hospitalized patients with COVID-19 to prevent the development of arterial and venous TEs [14,15,16]. Nowadays, this strategy has been demonstrated to be related to a reduction in mortality and TEs [17]. In fact, some studies advocate the use of therapeutic doses of LMWH, according to the results of clinical trials [18,19]. Furthermore, the risk of TEs also occurs after hospital discharge, when their presence is associated with hospital readmission and 90-day mortality [14].

After more than 2 years of attempting to better understand the dysregulation of the immune system and coagulopathy, clear evidence has demonstrated the increased risk of TEs. Multiple excellent reviews and strong hypotheses were formulated in this regard [8,11,12,20,21]. However, major descriptive studies only focused on TEs are limited. Moreover, they tend to present a low sample size, mainly centered on venous thromboembolism [16] and critically ill patients hospitalized in the ICU [13]. There is a paucity of extensive studies with a large follow-up and detailed characterization of not only venous but also arterial TEs including the time of diagnosis, the specific incidence rate over the course of the pandemic, the implications for previous anticoagulant treatments, or the possible use of D-dimer levels as an accurate biomarker for the detection of TEs [20]. In addition, a description according to the geographical area could be of interest, with scarce studies in the Spanish population [22].

Taking this into account, here we aim to perform a large retrospective study including all hospitalized COVID-19 patients in a tertiary hospital in Spain during the first 18 months to fully characterize all TEs. Our objective is to describe the type of TE, the specific location, the significance of thrombotic risk factors and laboratory values, and the relevance of in-hospital meters or the implications for in-hospital diagnosis of TEs compared to those at admission. Moreover, we also intend to delve into the potential use of D-dimer for TE detection in COVID-19.

## 2. Materials and Methods

### 2.1. Study Design and Patient Selection

A retrospective cohort study was performed. All patients admitted to the Hospital Clínico Universitario de Valladolid, Spain (a tertiary care center providing health care to 250,000 inhabitants) between 1 March 2020 and 31 August 2021 with a diagnosis of COVID-19 were included. In all patients included, SARS-CoV-2 infection was confirmed by polymerase chain reaction (PCR) of a nasopharyngeal sample.

Data were obtained from the records contained in the minimum basic dataset (MBDS) of the hospital. The medical records included sex, date of birth, dates of hospital admission and discharge, medical center, and diagnosis and procedure codes, in accordance with the International Classification of Diseases 10th Revision, Clinical Modification (ICD-10-CM). In the case of the data related to the D-dimer test, they were obtained at the time in the hospital’s central laboratory by using a HemosIL D-dimer HS 500 (Instrumentation Laboratory SpA, Milano, Italy; reference range: 0.1–500 ng/mL), following the manufacturer’s instructions. This information matched that of the MBDS of the National Hospital Data Surveillance System in Spain [23]. This study adhered to the STROBE guidelines for cohort studies [24].

### 2.2. Thrombosis Identification

**Figure 1:**
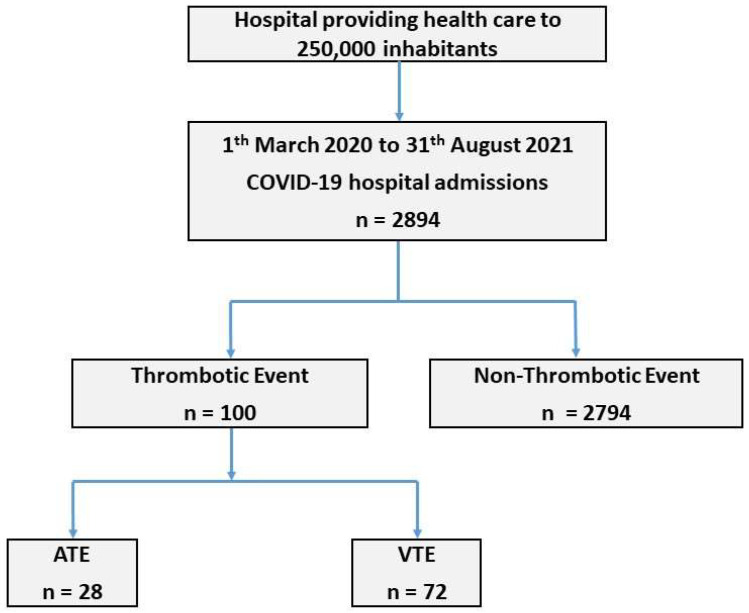
Flowchart of Patient Enrollment. ATE: arterial thrombotic event; VTE: venous thrombotic event.

The following ICD 10 codes (File S1) were used to identify all thrombotic events (TEs) in each of the COVID-19 patients admitted to the hospital [25]. The primary endpoint of the study was the presence of any venous (VTE) or arterial (ATE) thrombotic event at admission or during hospital stay. Primary or secondary diagnosis of TEs was also included. Subsequent confirmation was performed by reviewing the clinical record. Misdiagnoses, probable diagnoses of TEs without objective diagnostic proof, and non-thrombotic cerebral or cardiac infarctions were excluded (*n* = 39). Pulmonary embolism (PE) required a positive angiotomography, conventional angiography, or pulmonary perfusion scintigraphy. Deep vein thrombosis (DVT) required a compatible echo-doppler, ATE-associated compatible arterial echo-doppler, conventional arterial angiography, magnetic resonance imaging, computed tomography, or cardiac catheterization. Thus, 39 putative thrombotic diagnoses were misclassified and, thus, eliminated from the group of TEs. Assuming these criteria, our sample (*n* = 2894) was divided into two groups: (i) thrombotic event (*n* = 100) and (ii) non-thrombotic event (*n* = 2794). The flowchart in Figure 1 explains the study design and TE identification.

### 2.3. Thrombosis Characterization

Once the 100 TEs were correctly identified, a broad characterization was performed. The type of thrombotic event (venous or arterial), the specific localization, the presence of any antiaggregant or anticoagulant treatment at diagnosis, and the number of rethromboses were collected. In addition, risk factors and specific laboratory determinations related to thrombosis were included. Indeed, D-dimer plasma levels, platelet count, activated partial thromboplastin time (APTT), and prothrombin time (PT) at hospital admission and at the moment of the TE were included. Moreover, the moment of the TE was sought in detail to classify out-of-hospital (at admission) and in-hospital (during hospital stay) events. Patients were also categorized in terms of severity and mortality based on intensive care unit (ICU) admission, 90-day-mortality, or both (intubation or death risk). Comorbidity was defined by the Charlson index [26]. Finally, we complemented the patients’ descriptions together with the thrombophilia study, which was only performed in those patients with a direct request from the physician in charge. Factor V Leiden mutation, prothrombin G20210A mutation, protein C deficiency, protein S deficiency, antithrombin deficiency, coagulation factor VIII, Clauss fibrinogen, and lupus anticoagulant (dilute Russell’s viper venom (dRVV) and silica clotting time (SCT)) tests composed our routine thrombophilia study up to 65 years old; meanwhile, only coagulation factor VIII and lupus anticoagulant tests were conducted in the elderly.

### 2.4. Hospital Protocol Treatment

The hospital protocol for the treatment of COVID-19 pneumonia was changing as new evidence became available but included the following considerations: Lopinavir/Ritonavir 200/50 mg/mL solution twice a day and hydroxychloroquine 400 mg twice a day. Based on inflammatory criteria, the standard of care would also consider: Interferon 1β 0.25 mg every 48 h, corticosteroids 240 mg every day for three days, Tocilizumab, Baricitinib, or Anakinra. Antibiotic treatment was required in the case of suspected bacterial superinfection. Oxygen support (nasal cannula, high-flow nasal cannula, and non-invasive or invasive mechanical ventilation) was administered to patients depending on the severity of hypoxemia. All hospitalized patients received thrombotic prophylaxis using low-molecular-weight heparin (LMWH) with either bemiparin 3500 UI or enoxaparin 40 mg every day, which was maintained for 15 days after hospital discharge.

### 2.5. Statistical Analysis

In our population of 250,000 inhabitants, a sample size of 97 patients was calculated considering a confidence interval of 95% and a margin of error of 0.1. The sample size calculation was performed using the Piface software by Russel V. Lenth (version 1.76).

Descriptive statistics were used to summarize demographic data, clinical characteristics, and analytical data. Categorical variables were expressed as total number and percentage [*n* (%)], and significance was assessed by the chi-squared test. Continuous variables were represented by the median and interquartile range [median (IQR)], and significance was tested using Mann–Whitney U.

Multivariate regression models were performed regarding the evaluation of different associations: intubation or 90-day mortality risk and the presence of TEs; D-dimer levels and the identification of TEs; and D-dimer levels and mortality risk. Internal validation of each model was conducted with the leave-one-out-cross-validation (LOOCV) procedure and operation characteristic (ROC) curve analysis [27]. Cumulative event rate based on death or requirement of mechanical ventilation was determined using the Kaplan–Meier method by comparing D-dimer levels. Cumulative incidence curves were determined with the log-rank test. The stratified Cox proportional-hazards model was used to estimate the hazard ratio and 95% confidence interval.

Statistical analysis was performed by a PhD-licensed statistician using the R statistical package version 3.1.1 R Core Team and statistical package IBM SPSS Statistics software (SPSS) version 25. Statistical significance was fixed at *p* ≤ 0.05.

### 2.6. Ethics Approval

This study was approved by the Ethics Committee of the Hospital Clínico Universitario de Valladolid (cod: PI 20-1717) following the ethical code of the World Medical Association (Declaration of Helsinki). Confidentiality was adequately protected in accordance with Spanish data protection law.

## 3. Results

### 3.1. Comparison between TEs and Non-TEs

A total of 2894 patients were diagnosed with COVID-19 in our hospital area and were split into two groups: TE (*n* = 100) and non-TE (*n* = 2794). Clinical characteristics of both groups are shown in Table 1a. There were no differences between groups in terms of age. However, patients with TEs were on average older than 65 years old, with statistical significance (72 [72%] vs. 1715 [61.38%], *p* = 0.032). No differences were observed with regard to sex; however, males showed a tendency to be overrepresented in the TE group (61 [61%] vs. 1503 [53.8%], *p* = 0.155). In fact, the cumulative risk for TEs was markedly higher in males (log-rank = 4.601, *p* = 0.032), which is shown in Figure S1. Moreover, patients with TEs exhibited more comorbidities, calculated by a Charlson index ≥ 2 (29 [29%] vs. 565 [20.22%], *p* = 0.033), and were mainly associated with significantly higher rates of active cancer (13 [13%] vs. 107 [3.83%], *p* < 0.001) as well as higher domiciliary oxygen requirements at hospital discharge (18 [18%] vs. 320 [11.45%], *p* = 0.045). Finally, hospital meters revealed an increased length of hospital stay (12 [12] vs. 9 [9], p = 0.002), greater ICU admission requirement (25 [25%] vs. 303 [10.85%], *p* < 0.001), and higher intubation/90-day mortality risk (39 [39%] vs. 697 [24.94%], *p* = 0.002) in the TE group. A multivariate analysis was performed to evaluate the association between TEs and intubation or 90-day mortality risk in COVID-19, adjusted by gender, age > 65 years, and Charlson index ≥ 2 (Table S1). It was observed that the existence of any TE was associated with a 1.7-fold increased risk of intubation or 90-day mortality risk in COVID-19 (OR = 1.71, 95%CI (1.12–2.61), *p* < 0.013); likewise, being male, presenting age > 65 years, and a Charlson index ≥ 2 also increased the risk of poor outcome by a factor of 1.5–2.7.

### 3.2. Evolution of the Incidence Rate of TEs during the 18 Months of Follow-Up

**Figure 2:**
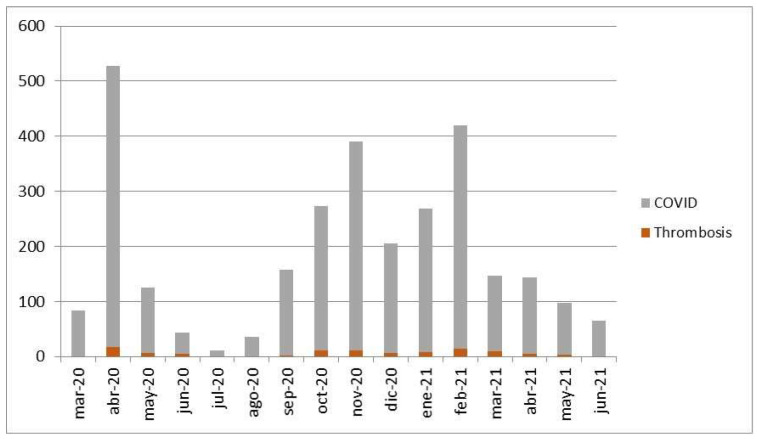
Evolution of the incidence rate of hospitalized thrombotic events (TEs) according to the incidence of in-hospital COVID-19 patients between 1 March 2020 and 31 August 2021.

For the follow-up period (1 March 2020 to 31 August 2021), the incidence rate of TEs month by month is shown in Figure 2. We were also able to observe the different dynamics throughout the waves of COVID-19 until the end of the fourth one in June 2021 in Spain. There were no new hospitalizations under the new diagnosis of COVID-19 or TEs in July and August 2021. The distribution of TEs along the different months of the study was distinct (*p* < 0.001), since TEs occurred just after the onset of a wave, with a consequent concomitant decrease in incidence.

### 3.3. Location of TE

The TE group was composed of 28 ATEs and 72 VTEs. Clinical characteristics of both groups are shown in Table 1b. There were no differences in terms of age and gender between ATEs and VTEs. The ATE group presented a higher prevalence of peripheral vascular disease (*p* = 0.023), while an increased requirement of domiciliary oxygen at hospital discharge (1 [3.67%] vs. 17 [23.61%], p = 0.019) was more frequent in the VTE group. Furthermore, D-dimer levels were significantly lower in the VTE group at admission (5649.5 [12,473] vs. 1404.5 [1617] ng/mL, p < 0.001) and at the time of the TE (7021 [15,633] vs. 2200 [4254] ng/mL, p < 0.001). No differences were found in relation to other laboratory measurements or prior antiplatelet or anticoagulant treatments. Hospital meters showed a higher requirement of ICU admission (13 [46.42%] vs. 12 [16.66%], p = 0.002) in COVID-19 patients with a diagnosis of an ATE, also associated with greater intubation/90-day mortality risk (16 [57.14%] vs. 23 [31.94%], *p* = 0.020).

**Figure 3:**
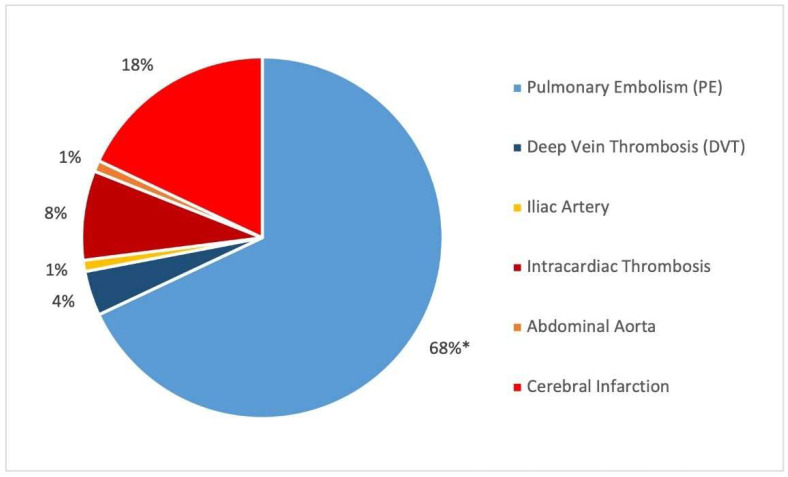
Location of the thrombotic events (*n* = 100). The venous ones (*n* = 72) are represented in blue, and the arterial ones (*n* = 28) are represented in orange-red; * means that the 68 cases with PE include 5 cases with DVT as well.

The specific location of each TE is represented in Figure 3. Most VTEs were pulmonary embolisms (68, 94.44%), and the remainder were deep vein thromboses. Five patients presented both VTE diagnoses at the same time. ATEs mainly corresponded to a diagnosis of cerebral infarction (18, 64.29%), followed by intracardiac thrombosis (8, 28.57%), iliac artery (1, 3.57%), and abdominal aorta (1, 3.57%).

**Figure 4:**
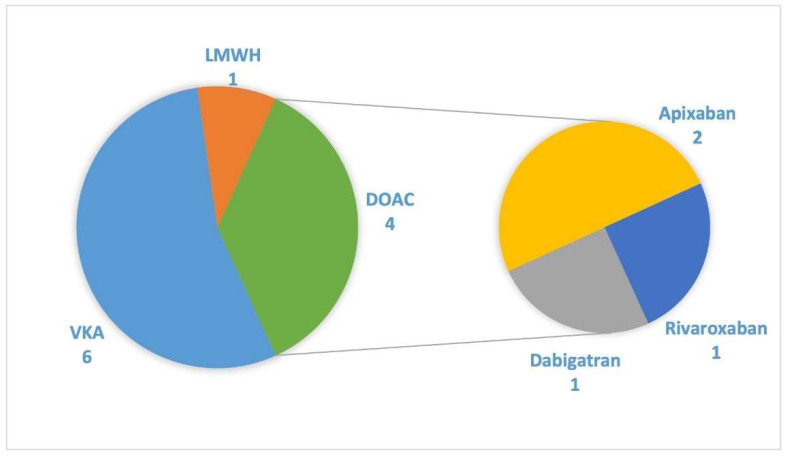
Type of anticoagulant therapy before admission in patients with domiciliary anticoagulant treatment (*n* = 11). VKA, vitamin K antagonists; LMWH; low-molecular-weight heparin; DOAC: direct oral anticoagulant (Apixaban 5 mg twice a day, Dabigatran 150 mg twice a day, and Rivaroxaban 20 mg once a day).

The presence of previous domiciliary anticoagulant therapy was observed in 11 patients (11% of TEs). Type of treatment is described in detail in Figure 4.

### 3.4. Importance of Out-Of-Hospital and In-Hospital Diagnosis of TEs

**Figure 5:**
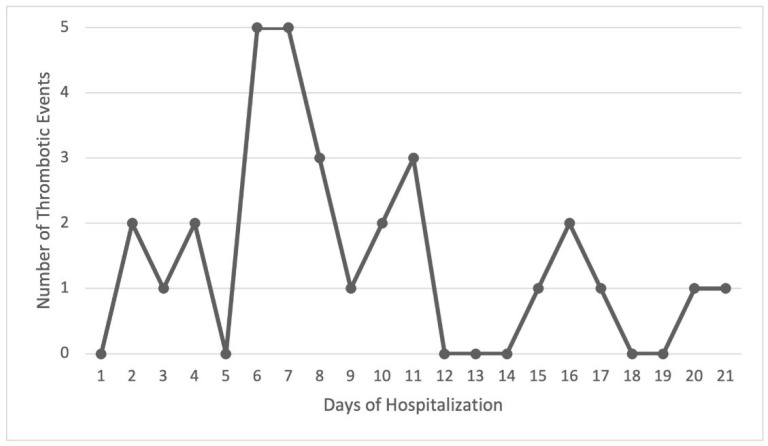
Day of in-hospital thrombotic event onset during hospital stay (*n* = 30).

Considering the 100 TEs, 70 of them (70%) were diagnosed at the moment of hospital admission, this being the reason for those patients attending the emergency department. Therefore, 70% of the TEs occurred in outpatients (out-of-hospital TEs). The remaining 30 events (30%) were diagnosed during the hospital stay (in-hospital TEs) in patients with no apparent thrombotic symptoms at admission. The day of diagnosis of these in-hospital TEs is described in Figure 5, occurring, in most cases, between days 6 and 11 of the hospital stay (19/30, 63.33%).

In-hospital TE diagnosis was significantly more frequent in males compared to out-of-hospital TEs (80% vs. 52.86%, *p* = 0.011). Moreover, previous antiplatelet therapy was predominant in the group of in-hospital thrombosis (8 [26.67%] vs. 5 [7.14%], *p* = 0.008). In-hospital TEs also presented significantly longer hospital (19 [21] vs. 9 [8], p < 0.001) and ICU stays (19 [28] vs. 5 [8], p = 0.050), but no differences in terms of mortality were found (Table 2a). The most interesting aspect for clinical practice may be the D-dimer levels at admission. Those COVID-19 patients who suffered TEs during their hospital stay showed a lower elevation of D-dimer levels at admission (918 [920] ng/mL) in comparison with reference values (up to 500 ng/mL). This presumably would be a consequence of the viral infection. However, those patients with a diagnosis of a TE at admission showed markedly higher levels (6272 [12,441] vs. 918 [920] ng/mL, *p* < 0.001) compared to those who did not have thrombosis at that moment.

The area under the receiver operating (ROC) curve revealed that D-dimer is an exceptional biomarker for detecting TEs in COVID-19 at admission (AUC = 0.91, *p* < 0.001) (Figure S2a). Furthermore, the resulting cut-off value of D-dimer was 2014 ng/mL, showing a sensitivity of 83% and a specificity of 80% (Table S2). An improved identification capability was observed considering only VTEs (AUC = 0.94, *p* < 0.001) (Figure S2b), with a cut-off value of 2666 pg/mL (sensitivity of 85% and specificity of 90%) (Table S2). A multivariate analysis was performed in which D-dimer levels over 2014 ng/mL at admission were associated with a higher risk of presenting any TE at that moment in the emergency department [OR = 16.78, 95%CI (5.21–54.01), *p* < 0.001]. In the case of VTEs, this risk was 77-fold higher when considering D-dimer levels over 2666 ng/mL [OR = 77.19, 95%CI (10.57–563.82), *p* < 0.001]. D-dimer, gender, age, active cancer, and prior antiplatelet and anticoagulant therapy were the variables included in both multivariate analyses (data not shown).

### 3.5. Mortality Associated with TEs in COVID-19

The comparison between survivors and non-survivors diagnosed with TE is shown in Table 2b. Non-survivors were predominantly male (18 [81.8%] vs. 43 [55.1%], *p* = 0.023) and of advanced aged (78 [13] vs. 72.5 [20], p = 0.032). Regarding comorbidities, differences were found only in alcohol consumption, which was higher in non-survivors (3 [13.6%] vs. 2 [2.56%], p = 0.035). Non-survivors presented significantly lower platelet count at both admission (207 [117.75] vs. 240 [161.5], p = 0.050) and the moment of TE diagnosis (214 [168.75] vs. 253 [181], p = 0.021), while D-dimer levels were extremely elevated at any time in these patients. However, there were no differences in either length of hospital stay or ICU admission.

In this scenario, D-dimer levels did not predict mortality in any TE (AUC = 0.67, *p* = 0.017) or in VTEs (AUC = 0.71, *p* = 0.011) (Table S3), as accurate as for identifying TEs at hospital admission. Kaplan–Meier curves in both any TE (Figure S3) and only VTEs (Figure S4) revealed a significantly higher cumulative percentage of non-survivors in those patients with D-dimer levels over its cut-off value of 8176 ng/mL (*p* = 0.003 and *p* = 0.005, respectively), in accordance with the results obtained from the stratified sex- and gender-adjusted Cox proportional-hazard models, which revealed that the presence of D-dimer levels over 8176 ng/mL was associated with a three-fold increased risk of 90-day mortality in any TE (hazard ratio: 3.03, CI 95% (1.26–7.26), *p* = 0.013), rising almost to a four-fold increased 90-day mortality risk in the case of VTEs only (hazard ratio: 3.83, CI 95% (1.22–12.08), *p* = 0.022) (Tables S4 and S5).

### 3.6. Thrombophilia Study

A total of 12 patients underwent a conventional thrombophilia study after hospital discharge. The median age was 54.5 years, five of them being over 65 years of age, and there were nine males and three females. The location of TEs corresponded to six VTEs (five PEs) and six ATEs (three cerebral infarctions, two intracardiac thromboses, and one iliac artery). The descriptive data regarding protein C, protein S, antithrombin, coagulation factor VIII, Clauss fibrinogen, and lupus anticoagulant (dRVV and SCT) tests were shown in Table 3 as well as the molecular biology test results for Factor V Leiden mutation and prothrombin G20210A mutation. The thrombophilia study was completely normal in all patients. There were no mutations, genetic deficiencies, or positive lupus anticoagulant testing. We could only highlight a slight elevation in coagulation factor VIII (199% [103]), in line with the inflammatory context of COVID-19.

## 4. Discussion

This retrospective study, including 2894 adult patients with COVID-19 who were admitted to the hospital and followed up over 18 months, is, to the best of our knowledge, the most complete study ever conducted in the Spanish population on both major arterial and venous thrombotic events. The most relevant findings of our investigation were: (i) the incidence of major thrombotic events (TEs) was 3.45%, involving a total of 100 events; (ii) the diagnosis of any TE was associated with a two-fold increased risk of intubation or 90-day mortality in COVID-19; (iii) venous thrombotic events (VTEs) were twice as frequent as arterial thrombotic events (ATEs), being related to higher levels of D-dimer; (iv) 70% of TEs were out-of-hospital TEs (occurring as outpatients and diagnosed at hospital admission), and the remaining 30% occurred during the hospital stay with LMWH-concomitant prophylaxis; (v) D-dimer levels above 2014 µg/mL at hospital admission identified TEs with an accuracy of 91%, rising to almost 95% with a cut-off value of 2666 µg/mL for identifying only VTEs; (vi) the top independent risk factor of mortality in TEs was, once again, D-dimer; (vii) the thrombophilia study was normal for all studied cases.

During these two pandemic years, after coagulopathy and prothrombotic state were suggested in COVID-19, studies evaluated the incidence of TEs in hospitalized patients. De vita et al. reported 121 events in 2579 COVID-19 patients (4.7%) with AIRD, of which one half were VTEs [28]. Ilyas et al. encountered a rate of 3.9% for ATEs and 3.7% for VTEs in a U.S. registry enrolling 21,528 hospitalized adults in which males were overrepresented [29]. Lodigiani et al. recorded 28 events (7.7%) in 388 Italian patients, most of them diagnosed at hospital admission [30]. Oba et al. studied 516 patients in Tokyo, of whom 42 patients (6.20%) suffered a TE [31]. Furthermore, Klok et al. described a TE incidence of 31% in ICU patients with COVID-19 [13]. Moreover, Giannis et al. reported that 1.55% of COVID-19 patients suffered a VTE within 90 days after hospital discharge [32]. In contrast, lower incidence rates of TEs (3.45%) were found in our study. These rates are closer to the results described in those studies with larger sample sizes. Every TE in our hospital database was verified in order to avoid misdiagnosis, such as erroneous over-diagnosis, especially associated with ATE. Therefore, the incidence rate in the Spanish population might be slightly lower than that of other population-based studies; however, prospective and robust studies should be performed.

The occurrence of thrombotic events is more frequently associated with advanced age, male sex, and the presence of TE risk factors [14,30,31]. These are essentially the same factors that are associated with severe COVID-19 [1,5,33,34]. Nevertheless, we observed a higher Charlson index, older age, and a higher proportion of males as well as poorer in-hospital meters in the TE group. The greater comorbidity related to the presence of TEs in patients with COVID-19 compared to those without TEs would render this type of patient particularly vulnerable. Although these differences have already been described, the timing of clinical presentation could possibly be the most interesting finding of our investigation. In fact, 70% of the TEs in our work were diagnosed at hospital admission (out-of-hospital TE), including 11 patients with domiciliary anticoagulation therapy. This implies that 41% of TEs occurred in patients who were receiving LMWH thromboprophylactic treatments in hospital or domiciliary therapies. Most of the studies are focused on debating anticoagulation doses and the length of time for which antithrombotic therapy should be sustained after hospital discharge [35,36]. As far as we know, it was only Lodigiani et al. who reported that 50% of TEs were diagnosed after hospital admission [30]. This deserves some reflection and consideration of what this is due to and which strategies we could implement to prevent thrombosis. For example, recent clinical trials advocate the use of therapeutic doses of LMWH [18,19]. If we analyze the incidence rates month by month, TEs are as frequent as they were at the beginning of the pandemic. The sedentary lifestyle derived from the confinement imposed by the pandemic in 2020 for weeks and even months might have an influence on this [37], being a key point in explaining the increased number of TEs, especially during the first wave. Moreover, as we showed, the more comorbidity a patient exhibits, the higher is the risk of severe COVID-19 and TEs.

With regard to thrombotic predisposition in COVID-19, autopsies identified unsuspected thromboembolism or microthrombosis in alveolar capillaries in over 60% of patients [38]. This supports the existence of a hypercoagulability state, the underlying mechanisms of which are still unclear. It is presumed to be influenced by different interactions [39] involving the immune system (cytokine release), the inflammatory system (mediators of inflammation), and the coagulation systems [8,40]. Moreover, increased platelet activity related to viral-mediated endothelial inflammation appears to be implicated [41]. D-dimer (cross-linked fibrin derivatives formed during thrombolysis) is the most commonly associated laboratory biomarker of TEs in COVID-19-related hypercoagulability [20]. Several studies have described that increased plasma levels of D-dimer predict risk of TEs and mortality [42,43], as we evidenced in our work. The negative predictive value of D-dimer in TEs is widely known [44], but its levels are not used to identify TEs (positive predictive value), especially when baseline D-dimer levels are already elevated, for example, in COVID-19. Furthermore, the routine COVID-19 analytical profile generally includes D-dimer, making it impossible to use pre-test probability (low, intermediate, high) in VTE screening. Thus, the positive predictive value of D-dimer would be even more useful. In our study, we observed that presentation of plasma levels of D-dimer above 2014 µg/mL at hospital admission identifies TEs with 90% accuracy, rising to almost 95% with a cut-off value of 2666 µg/mL for identifying only VTEs. Some studies supported our results, demonstrating statistically significant results when comparing D-dimer levels in TE and non-TE patients with COVID-19 [31,45,46,47]. The principal differences with respect to these studies reside in the cut-off values that we proposed. Thus, and in accordance with our results, increased D-dimer levels above 2014 µg/mL for any TE or 2666 µg/mL for VTEs could be sufficient reason to conduct diagnostic radiological testing for thrombosis detection. Therefore, besides reference intervals and cut-offs, D-dimer levels have very specific applications (e.g., disseminated intravascular coagulation (DIC)); COVID-19 would be another entity to consider.

A further important point on which we focused was thrombophilia. The majority of studies did not report significant alterations in routine coagulation tests (APTT and PT), in line with our results [31,47]. Platelet count might be related to prognosis, decreasing in severe and late phases of the disease [31,48]. A number of studies profiled natural anticoagulants, coagulant factors, and anti-phospholipid antibodies during hospital stay, especially in critically ill patients [49], showing values of protein C, protein S, antithrombin, and coagulation factors V and VII below the normal range, while coagulation factor VIII activities were significantly above the normal range [49]. However, these abnormalities are plausible in any severe infectious or acute thrombotic process. The thrombophilia study should be performed after the acute phase, with no studies available at the present moment. In our work, considering the scant number of patients with a thrombophilia study, no relevant alterations were detected, with the exception of a minimal persistent elevation in coagulation factor VIII activity. This could explain a delayed recovery from viral infection and the associated increase in D-dimer levels in some patients after two months. These considerations confirm the existence of a COVID-19-related coagulopathy, this dysregulation being directly responsible for TEs (not influenced by a positive thrombophilia study).

Finally, we should point out some limitations of this study. Firstly, it is a retrospective database-driven design. Secondly, no routine radiological screening test was performed to identify thromboembolism, which may underestimate the diagnoses of thrombosis. Thirdly, there was a small sample size in the TE group. Thirdly, the results obtained with respect to D-dimer as a biomarker for detecting TEs in COVID-19 at admission must be clinically validated in other prospective multicenter cohorts. Lastly, this is a single-center study, usually an obvious limitation, but here we were able to obtain complete and optimal information from the clinical records to better characterize every patient and attempt to obtain the optimum results.

## 5. Conclusions

The incidence of major thrombotic events (TEs) was 3.45%, mainly in elderly males with comorbidities and increasing two-fold the risk of intubation or 90-day mortality in COVID-19. Venous thrombotic events (VTEs) were clearly more frequent than arterial thrombotic events (ATE), associated with higher D-dimer levels. 70% of the diagnoses were out-of-hospital TEs, and almost 50% of TEs received concomitant anticoagulant treatment (prophylaxis with LMWH in hospital or anticoagulant therapy at home). Our study found an association between D-dimer levels and early TE identification in COVID-19 (positive predictive value); however, further prospective studies should be conducted to validate these results and find the best cut-off value from which to perform a diagnostic radiological test. Normal thrombophilia study findings after the acute process, except for a minimal persistent elevation of coagulation factor VIII activity, are in accordance with an independent procoagulant state and SARS-CoV-2-derived coagulopathy. These considerations would have a direct benefit in clinical practice in terms of knowing how and when we should identify a TE and also for the development of new appropriate therapeutic and prophylactic approaches.

## Figures and Tables

**Table 1:** (a) Clinical characteristics of hospitalized COVID-19 patients according to the diagnosis of thrombotic events (TE). (b) Different characteristics between arterial and venous thrombotic events.

	(a) Presence of Thrombotic Event (TE)			(b) Type of Thrombotic Event		
	TE (*n* = 100)	Non-TE (*n* = 2794)	*p*	ATE (*n* = 28)	VTE (*n* = 72)	*p*
**Age**						
Age, [median [IQR]]	73.50 [21]	71 [24]	0.190	71 [21]	74 [21]	0.511
Age > 65 years, [*n* (%)]	72 (72)	1715 (61.38)	**0.032**	20 (71.43)	52 (72.22)	0.937
**Sex, [*n* (%)]**						
Female	39 (39)	1291 (46.20)	0.155	9 (32.14)	30 (41.67)	0.381
Male	61 (61)	1503 (53.80)		19 (67.85)	42 (58.33)	
**Comorbidities, [*n* (%)]**						
Previous TE	-	-	-	7 (25)	8 (11.11)	0.081
Charlson index ≥ 2	29 (29)	565 (20.22)	**0.033**	7 (25)	22 (30.56)	0.583
Hypertension	49 (49)	1271 (45.49)	0.489	16 (57.14)	33 (45.83)	0.310
Smoking	23 (23)	609 (21.80)	0.775	10 (35.71)	13 (18.05)	0.06
Diabetes mellitus	23 (23)	551 (19.72)	0.419	10 (35.71)	13 (18.05)	0.06
Dyslipemia	37 (37)	858 (30.71)	0.181	9 (32.14)	28 (38.88)	0.53
Atrial fibrillation	8 (8)	227 (8.12)	0.964	3 (10.71)	5 (6.94)	0.53
Coronary heart disease	2 (2)	132 (4.72)	0.203	0 (0)	2 (2.77)	0.373
Cardiac disease	4 (4)	103 (3.69)	0.870	1 (3.5)	3 (4.16)	0.892
Chronic kidney disease	10 (10)	246 (8.8)	0.679	3 (10.71)	7 (9.78)	0.882
Pulmonary disease	9 (9)	285 (10.20)	0.696	3 (10.71)	6 (8.33)	0.709
Domiciliary oxygen at discharge	18 (18)	320 (11.45)	**0.045**	1 (3.67)	17 (23.61)	**0.019**
Peripheral vascular disease	8 (8)	176 (6.30)	0.493	5 (17.85)	3 (4.16)	**0.023**
Active cancer	13 (13)	107 (3.83)	**<0.001**	1 (3.67)	12 (16.66)	0.08
TE at admission	-	-	-	17 (60.71)	53 (77.61)	0.206
**Pre-admission pharmacological treatments, [*n* (%)]**						
Antiaggregant therapy	-	-	-	4 (14.28)	9 (12.5)	0.812
Anticoagulant therapy	-	-	-	4 (14.28)	7 (9.72)	0.513
**Laboratory values, [median [IQR]]**						
Platelet at admission, (×10^3^/L)	-	-	-	262 [124.5]	210 [152.25]	0.116
Platelet at TE onset, (×10^3^/L)	-	-	-	290 [130.25]	227.50 [178.5]	0.203
D-dimer at admission, (ng/mL)	-	-	-	1404.5 [1617]	5649 [12,473]	**<0.001**
D-dimer at TE onset, (ng/mL)	-	-	-	2200 [4254]	7021 [15,633]	**<0.001**
D-dimer after 2 months, (ng/mL)		-	-	379 [736.50]	331.50 [332.5]	0.525
APTT at admission (s)	-	-	-	27.5 [5]	27 [4]	0.593
PT at admission, (s)	-	-	-	13.5 [1.25]	13 [3]	0.989
**Hospital meters**						
Length of hospital stay, days [median [IQR]]	12 [12]	9 [9]	**0.002**	12.5 [21]	12 [10]	0.298
ICU admission, [*n* (%)]	25 (25)	303 (10.85)	**<0.001**	13 (46.42)	12 (16.66)	**0.002**
Length of ICU stay, days [median [IQR]]	8 [15]	15 [20]	**0.021**	4 [21]	11.5 [15]	0.219
90-day mortality, [*n* (%)]	22 (22)	501 (17.93)	0.299	6 (21.42)	16 (22.22)	0.931
Intubation/90-day mortality, [*n* (%)]	39 (39)	697 (24.94)	**0.002**	16 (57.14)	23 (31.94)	**0.020**

Continuous variables are represented as [median, (interquartile range, IQR)]; categorical variables are represented as [*n*, (%)]. TE, thrombotic event; ATE, arterial thrombotic event; VTE, venous thrombotic event; D-dimer, dimerized plasmin fragment D; PT, prothrombin time; APTT, activated partial thromboplastin time.

**Table 2:** (a): Clinical characteristics comparing out-of-hospital thrombotic events (diagnosis at hospital admission) and in-hospital thrombotic events (in-hospital diagnosis). (b) Differences in terms of mortality according to the presence of thrombotic events.

	(a) Type of TE			(b) Mortality According to TE		
	Out-Of-Hospital (*n* = 70)	In-Hospital (*n* = 30)	*p*	Survivors (*n* = 78)	Non-Survivors (*n* = 22)	*p*
**Age**						
Age, [median [IQR]]	73.50 [20]	73.7 [23]	0.916	72.5 [20]	78 [13]	**0.032**
Age > 65 years, [*n* (%)]	50 (71.43)	22 (73.33)	0.846	53 (67.95)	19 (86.36)	0.089
**Sex, [*n* (%)]**						
Female	33 (47.14)	6 (20)	**0.011**	35 (44.9)	4 (18.2)	**0.023**
Male	37 (52.86)	24 (80)		43 (55.1)	18 (81.8)	
**Comorbidities, [*n* (%)]**						
Previous TE	9 (12.86)	6 (20)	0.359	11 (14.1)	4 (18.18)	0.636
Charlson index ≥ 2	20 (28.57)	9 (30)	0.885	20 (25.64)	9 (40.91)	0.163
Hypertension	34 (48.55)	15 (50)	0.896	37 (47.4)	12 (54.54)	0.556
Smoking	15 (21.43)	8 (26.67)	0.568	16 (20.5)	7 (31.8)	0.266
Alcohol consumption	3 (4.29)	2 (6.67)	0.617	2 (2.56)	3 (13.6)	**0.035**
Diabetes mellitus	14 (20)	9 (30)	0.276	16 (20.51)	7 (31.81)	0.266
Dislipemia	29 (41.43)	8 (26.67)	0.161	30 (38.46)	7 (31.81)	0.569
Atrial fibrillation	5 (7.14)	3 (10)	0.629	6 (7.69)	2 (9.09)	0.831
Coronary heart disease	1 (1.43)	1 (3.33)	0.533	1 (1.28)	1 (4.54)	0.334
Cardiac disease	2 (2.86)	2 (6.67)	0.373	3 (3.84)	1 (4.54)	0.882
Chronic kidney disease	5 (7.14)	5 (16.67)	0.146	8 (10.25)	2 (9.09)	0.872
Pulmonary disease	7 (10)	2 (6.67)	0.594	7 (8.97)	2 (9.09)	0.987
Domiciliary oxygen at discharge	12 (17.14)	6 (20)	0.733	-	-	-
Peripheral vascular disease	5 (7.14)	3 (10)	0.629	6 (7.69)	2 (9.09)	0.831
Active cancer	12 (17.14)	1(3.33)	0.060	9 (11.53)	4 (18.18)	0.413
**Characteristics of the TE, [*n* (%)]**						
ATE	17 (24.28)	11 (36.67)	0.206	22 (28)	6 (27.2)	0.931
TE at admission	-	-	-	54 (69.23)	16 (72.73)	0.752
**Pre-admission pharmacological treatments, [*n* (%)]**						
Antiaggregant therapy	5 (7.14)	8 (26.67)	**0.008**	9 (11.53)	4 (18.18)	0.413
Anticoagulant therapy	7 (10)	4 (13.3)	0.625	9 (11.53)	3 (13.63)	0.789
**Laboratory values, [median [IQR]]**						
Platelet at admission, (×10^3^/L)	249 [176]	203 [100.5]	0.037	240 [161.5]	207 [117.75]	**0.050**
Platelet at TE, (×10^3^/L)	249 [176]	230 [189]	0.758	253 [181]	214 [168.75]	**0.021**
D-dimer at admission, (ng/mL)	6272 [12,441]	918 [920]	**<0.001**	2755.5 [5702]	6312 [17,597]	0.096
D-dimer at TE, (ng/mL)	6272 [12,441]	5432 [22,144]	0.604	5536 [9816]	13863 [39,605]	**0.017**
D-dimer after 2 months, (ng/mL)	336 [367]	384 [1112]	0.532	336 [326]	1530 [1451]	0.09 *
**Hospital meters**						
Length of hospital stay, days [median (IQR)]	9 [8]	19 [21]	**<0.001**	11.5 [13]	13 [14]	0.990
ICU admission, [*n* (%)]	13 (18.57)	12 (40)	**0.023**	17 (21.8)	8 (36.3)	0.173
Length of ICU stay, days [median (IQR)]	5 [8]	19 [28]	**0.050**	6 [15]	16 [29]	0.412
90-day-mortality, [*n* (%)]	16 (22.86)	6(20)	0.752	-	-	-
Intubation/death, [*n* (%)]	25 (35.71)	14 (46.67)	0.303	-	-	-

Continuous variables are represented as [median, (interquartile range, IQR)]; categorical variables are represented as [%, (*n*)]. TE, thrombotic event; ATE, arterial thrombotic event; VTE, venous thrombotic event; D-dimer, dimerized plasmin fragment D; PT, prothrombin time; APTT, activated partial thromboplastin time; * only four patients composed the group of non-survivors.

**Table 3:** Description of the results of the thrombophilia study (*n* = 12) performed after acute viral infection.

Thrombophilia Study	
dRVV, [median (IQR)]	1.12 (0.56)
SCT, [median (IQR)]	1.13 (0.36)
Protein S, (%), [median (IQR)]	73.5 (41)
Protein C, (%), [median (IQR)]	108.5 (35)
Coagulation factor VIII, (%), [median (IQR)]	**199 (103)**
Antithrombin, (%), [median (IQR)]	98 (33)
Clauss fibrinogen, (mg/dL), [median (IQR)]	363 (363)
Factor V Leiden mutation, [*n* (%)]	12, 100% (No mutated)
Prothrombin G20210A mutation, [*n* (%)]	12, 100% (No mutated)

Continuous variables are represented as [median, (interquartile range, IQR)]; categorical variables are represented as [*n* (%)]. dRRV, dilute Russell’s viper venom time; SCT, silica clotting time.

## Data Availability

The data presented in this study are available on request from the corresponding author.

## Supplementary Materials

The following supporting information can be downloaded at: https://www.mdpi.com/article/10.3390/jcm11123443/s1, File S1. ICD 10 codes used to identify all thrombotic events (TEs) in each of the COVID-19 patients admitted to the hospital; Figure S1. Cumulative risk for TEs depending on gender; Figure S2. (a) ROC curve analysis for evaluating D-dimer accuracy to detect TEs in COVID-19 at admission. (b) ROC curve analysis for evaluating D-dimer accuracy to detect VTEs in COVID-19 at admission; Figure S3. Kaplan–Meier survival curves in TEs for 90-day mortality; Figure S4. Kaplan–Meier survival curves in VTEs for 90-day mortality; Table S1. Multivariate analysis for evaluating the association between TE and risk of intubation or 90-day mortality in COVID-19; Table S2. Accuracy of D-dimer levels for detecting all TEs and VTEs; Table S3. D-dimer level accuracy for detecting mortality in all TEs and in VTEs only; Table S4. Stratified Cox proportional-hazard model for 90-day mortality in any TE; Table S5. Stratified Cox proportional-hazard model for 90-day mortality in VTE.
