# Supplementary material for: Full Characterization of Thrombotic Events in All Hospitalized COVID-19 Patients in a Spanish Tertiary Hospital during the First 18 Months of the Pandemic
Source: J Clin Med. 2022 Jun 15;11(12):3443. doi: 10.3390/jcm11123443 (PMC9225147; doi:10.3390/jcm11123443)
Supplement: Supplementary file 1 [file jcm-11-03443-s001.zip › File S1.pdf]

ICD-10 Codes used for diagnosis of arterial and venous TE

Venous thrombotic events:

I26 Pulmonary embolism

- I26.0 Pulmonary embolism with acute cor pulmonale
    - I26.02 Saddle embolus of pulmonary artery with acute cor pulmonale
    - I26.09 Other pulmonary embolisms with acute cor pulmonale
- I26.9 Pulmonary embolisms without acute cor pulmonale
    - I26.92 Saddle embolus of pulmonary artery without acute cor pulmonale
    - I26.93 Single subsegmental pulmonary embolism without acute cor pulmonale
    - I26.94 Multiple subsegmental pulmonary emboli without acute cor pulmonale
    - I26.99 Other pulmonary embolisms without acute cor pulmonale

I82 Other venous embolism and thrombosis

- I81 Portal vein thrombosis
- I82.0 Budd-Chiari syndrome
- I82.3 Embolism and thrombosis of renal vein
- I82.4 Acute embolism and thrombosis of deep veins of lower extremity
    - I82.40 Acute embolism and thrombosis of unspecified deep veins of lower extremity
        - I82.401 Acute embolism and thrombosis of unspecified deep veins of right lower extremity
        - I82.402 Acute embolism and thrombosis of unspecified deep veins of left lower extremity
        - I82.403 Acute embolism and thrombosis of unspecified deep veins of lower extremities, bilateral
        - I82.409 Acute embolism and thrombosis of unspecified deep veins of unspecified lower extremity
    - I82.41 Acute embolism and thrombosis of femoral vein
        - I82.411 Acute embolism and thrombosis of right femoral vein
        - I82.412 Acute embolism and thrombosis of left femoral vein
        - I82.413 Acute embolism and thrombosis of femoral veins, bilateral
        - I82.419 Acute embolism and thrombosis of unspecified femoral vein
    - I82.42 Acute embolism and thrombosis of iliac vein
        - I82.421 Acute embolism and thrombosis of right iliac vein
        - I82.422 Acute embolism and thrombosis of left iliac vein
        - I82.423 Acute embolism and thrombosis of iliac veins, bilateral
        - I82.429 Acute embolism and thrombosis of unspecified iliac vein
    - I82.43 Acute embolism and thrombosis of popliteal vein
        - I82.431 Acute embolism and thrombosis of right popliteal vein
        - I82.432 Acute embolism and thrombosis of left popliteal vein
        - I82.433 Acute embolism and thrombosis of popliteal veins, bilateral
        - I82.439 Acute embolism and thrombosis of unspecified popliteal vein
    - I82.44 Acute embolism and thrombosis of tibial vein
        - I82.441 Acute embolism and thrombosis of right tibial vein
        - I82.442 Acute embolism and thrombosis of left tibial vein

        - I82.443 Acute embolism and thrombosis of tibial veins, bilateral
        - I82.449 Acute embolism and thrombosis of unspecified tibial vein
    - I82.45 Acute embolism and thrombosis of peroneal vein
        - I82.451 Acute embolism and thrombosis of right peroneal vein
        - I82.452 Acute embolism and thrombosis of left peroneal vein
        - I82.453 Acute embolism and thrombosis of peroneal veins, bilateral
        - I82.459 Acute embolism and thrombosis of unspecified peroneal vein
    - I82.46 Acute embolism and thrombosis of calf muscular vein
        - I82.461 Acute embolism and thrombosis of right calf muscular vein
        - I82.462 Acute embolism and thrombosis of left calf muscular vein
        - I82.463 Acute embolism and thrombosis of calf muscular veins, bilateral
        - I82.469 Acute embolism and thrombosis of unspecified calf muscular vein
    - I82.49 Acute embolism and thrombosis of other specified deep vein of lower extremity
        - I82.491 Acute embolism and thrombosis of other specified deep vein of right lower extremity
        - I82.492 Acute embolism and thrombosis of other specified deep vein of left lower extremity
        - I82.493 Acute embolism and thrombosis of other specified deep vein of lower extremities, bilateral
        - I82.499 Acute embolism and thrombosis of other specified deep vein of unspecified lower extremity
    - I82.4Y Acute embolism and thrombosis of unspecified deep veins of proximal lower extremity
        - I82.4Y1 Acute embolism and thrombosis of unspecified deep veins of right proximal lower extremity
        - I82.4Y2 Acute embolism and thrombosis of unspecified deep veins of left proximal lower extremity
        - I82.4Y3 Acute embolism and thrombosis of unspecified deep veins of proximal lower extremities, bilateral
        - I82.4Y9 Acute embolism and thrombosis of unspecified deep veins of unspecified proximal lower extremity
    - I82.4Z Acute embolism and thrombosis of unspecified deep veins of distal lower extremity
        - I82.4Z1 Acute embolism and thrombosis of unspecified deep veins of right distal lower extremity
        - I82.4Z2 Acute embolism and thrombosis of unspecified deep veins of left distal lower extremity
        - I82.4Z3 Acute embolism and thrombosis of unspecified deep veins of distal lower extremities, bilateral
        - I82.4Z9 Acute embolism and thrombosis of unspecified deep veins of unspecified distal lower extremity
- I82.6 Acute embolism and thrombosis of veins of upper extremity
    - I82.62 Acute embolism and thrombosis of deep veins of upper extremity
        - I82.621 Acute embolism and thrombosis of deep veins of right upper extremity
        - I82.622 Acute embolism and thrombosis of deep veins of left upper

        extremity
        - I82.623 Acute embolism and thrombosis of deep veins of upper extremities, bilateral
        - I82.629 Acute embolism and thrombosis of deep veins of unspecified upper extremity
- I82.A Embolism and thrombosis of axillary vein
    - I82.A1 Acute embolism and thrombosis of axillary vein
        - I82.A11 Acute embolism and thrombosis of right axillary vein
        - I82.A12 Acute embolism and thrombosis of left axillary vein
        - I82.A13 Acute embolism and thrombosis of axillary veins, bilateral
        - I82.A19 Acute embolism and thrombosis of unspecified axillary vein
- I82.B Embolism and thrombosis of subclavian vein
    - I82.B1 Acute embolism and thrombosis of subclavian vein
        - I82.B11 Acute embolism and thrombosis of right subclavian vein
        - I82.B12 Acute embolism and thrombosis of left subclavian vein
        - I82.B13 Acute embolism and thrombosis of subclavian veins, bilateral
        - I82.B19 Acute embolism and thrombosis of unspecified subclavian vein
- I82.8 Embolism and thrombosis of other specified veins
    - I82.81 Embolism and thrombosis of superficial veins of lower extremities
        - I82.811 Embolism and thrombosis of superficial veins of right lower extremity
        - I82.812 Embolism and thrombosis of superficial veins of left lower extremity
        - I82.813 Embolism and thrombosis of superficial veins of lower extremities, bilateral
        - I82.819 Embolism and thrombosis of superficial veins of unspecified lower extremity

Arterial thrombotic events:

G45 Transient cerebral ischemic attacks and related syndromes

- G45.0 Vertebro-basilar artery syndrome
- G45.1 Carotid artery syndrome (hemispheric)
- G45.2 Multiple and bilateral precerebral artery syndromes
- G45.3 Amaurosis fugax
- G45.4 Transient global amnesia
- G45.8 Other transient cerebral ischemic attacks and related syndromes
- G45.9 Transient cerebral ischemic attack, unspecified

I20 Angina pectoris

- I20.0 Unstable angina

I21 Acute myocardial infarction

- I21.0 ST elevation (STEMI) myocardial infarction of anterior wall
    - I21.01 ST elevation (STEMI) myocardial infarction involving left main coronary artery
    - I21.02 ST elevation (STEMI) myocardial infarction involving left anterior descending coronary artery
    - I21.09 ST elevation (STEMI) myocardial infarction involving other coronary artery of anterior wall
- I21.1 ST elevation (STEMI) myocardial infarction of inferior wall

    - I21.11 ST elevation (STEMI) myocardial infarction involving right coronary artery
    - I21.19 ST elevation (STEMI) myocardial infarction involving other coronary artery of inferior wall
- I21.2 ST elevation (STEMI) myocardial infarction of other sites
    - I21.21 ST elevation (STEMI) myocardial infarction involving left circumflex coronary artery
    - I21.29 ST elevation (STEMI) myocardial infarction involving other sites
- I21.3 ST elevation (STEMI) myocardial infarction of unspecified site
- I21.4 Non-ST elevation (NSTEMI) myocardial infarction
- I21.9 Acute myocardial infarction, unspecified

I22 Subsequent ST elevation (STEMI) and non-ST elevation (NSTEMI) myocardial infarction

- I22.0 Subsequent ST elevation (STEMI) myocardial infarction of anterior wall
- I22.1 Subsequent ST elevation (STEMI) myocardial infarction of inferior wall
- I22.2 Subsequent non-ST elevation (NSTEMI) myocardial infarction
- I22.8 Subsequent ST elevation (STEMI) myocardial infarction of other sites
- I22.9 Subsequent ST elevation (STEMI) myocardial infarction of unspecified site

I23 Certain current complications following ST elevation (STEMI) and non-ST elevation (NSTEMI) myocardial infarction (within the 28 day period)

- I23.6 Thrombosis of atrium, auricular appendage, and ventricle as current complications following acute myocardial infarction

I24 Other acute ischemic heart diseases

- I24.0 Acute coronary thrombosis not resulting in myocardial infarction
- I24.8 Other forms of acute ischemic heart disease
- I24.9 Acute ischemic heart disease, unspecified

I51.3 Intracardiac thrombosis, not elsewhere classified

I63 Cerebral infarction

- I63.0 Cerebral infarction due to thrombosis of precerebral arteries
    - I63.00 Cerebral infarction due to thrombosis of unspecified precerebral artery
    - I63.01 Cerebral infarction due to thrombosis of vertebral artery
        - I63.011 Cerebral infarction due to thrombosis of right vertebral artery
        - I63.012 Cerebral infarction due to thrombosis of left vertebral artery
        - I63.013 Cerebral infarction due to thrombosis of bilateral vertebral arteries
        - I63.019 Cerebral infarction due to thrombosis of unspecified vertebral artery
    - I63.02 Cerebral infarction due to thrombosis of basilar artery
    - I63.03 Cerebral infarction due to thrombosis of carotid artery
        - I63.031 Cerebral infarction due to thrombosis of right carotid artery
        - I63.032 Cerebral infarction due to thrombosis of left carotid artery
        - I63.033 Cerebral infarction due to thrombosis of bilateral carotid arteries
        - I63.039 Cerebral infarction due to thrombosis of unspecified carotid artery
    - I63.09 Cerebral infarction due to thrombosis of other precerebral artery
- I63.3 Cerebral infarction due to thrombosis of cerebral arteries
    - I63.30 Cerebral infarction due to thrombosis of unspecified cerebral artery
    - I63.31 Cerebral infarction due to thrombosis of middle cerebral artery
        - I63.311 Cerebral infarction due to thrombosis of right middle cerebral

artery
        - I63.312 Cerebral infarction due to thrombosis of left middle cerebral artery
        - I63.313 Cerebral infarction due to thrombosis of bilateral middle cerebral arteries
        - I63.319 Cerebral infarction due to thrombosis of unspecified middle cerebral artery
    - I63.32 Cerebral infarction due to thrombosis of anterior cerebral artery
        - I63.321 Cerebral infarction due to thrombosis of right anterior cerebral artery
        - I63.322 Cerebral infarction due to thrombosis of left anterior cerebral artery
        - I63.323 Cerebral infarction due to thrombosis of bilateral anterior cerebral arteries
        - I63.329 Cerebral infarction due to thrombosis of unspecified anterior cerebral artery
    - I63.33 Cerebral infarction due to thrombosis of posterior cerebral artery
        - I63.331 Cerebral infarction due to thrombosis of right posterior cerebral artery
        - I63.332 Cerebral infarction due to thrombosis of left posterior cerebral artery
        - I63.333 Cerebral infarction due to thrombosis of bilateral posterior cerebral arteries
        - I63.339 Cerebral infarction due to thrombosis of unspecified posterior cerebral artery
    - I63.34 Cerebral infarction due to thrombosis of cerebellar artery
        - I63.341 Cerebral infarction due to thrombosis of right cerebellar artery
        - I63.342 Cerebral infarction due to thrombosis of left cerebellar artery
        - I63.343 Cerebral infarction due to thrombosis of bilateral cerebellar arteries
        - I63.349 Cerebral infarction due to thrombosis of unspecified cerebellar artery
    - I63.39 Cerebral infarction due to thrombosis of other cerebral artery

I74 Arterial embolism and thrombosis

- I74.2 Embolism and thrombosis of arteries of the upper extremities
- I74.3 Embolism and thrombosis of arteries of the lower extremities
- I74.4 Embolism and thrombosis of arteries of extremities, unspecified
- I74.5 Embolism and thrombosis of iliac artery
