# Supplementary material for: Full Characterization of Thrombotic Events in All Hospitalized COVID-19 Patients in a Spanish Tertiary Hospital during the First 18 Months of the Pandemic
Source: J Clin Med. 2022 Jun 15;11(12):3443. doi: 10.3390/jcm11123443 (PMC9225147; doi:10.3390/jcm11123443)
Supplement: Supplementary file 1 [file jcm-11-03443-s001.zip › jcm-1680135-supplementary.pdf]

Journal of Clinical Medicine

Table S1. Multivariate Analysis for evaluating the association between TE and risk of intubation or 90-day mortality in COVID-19 disease.

	OR	95% CI	*p*
**TE**	**1.71**	1.12-2.61	**0.013**
Sex	1.61	1.35-1.92	**<0.001**
Age > 65 years old	2.73	2.23-3.34	**<0.001**
Charlson Index ≥ 2	1.39	1.13-1.70	**0.002**

TE, thrombotic event; CI, confidence interval; OR, odds ratio.

Table S2. Accuracy of D-Dimer levels for detecting all TE events and VTE events.

Type of TE	AUC	*p*	Cut-off value	Sensitivity	Specificity
**All TE events**	0.91	<0.001	2014 ng/mL	83%	80%
**VTE Events**	0.94	<0.001	2666 ng/mL	85%	90%

Table S3. D Dimer levels accuracy for detecting mortality in all TE events and only in VTE events.

Biomarker	AUC	*p*	Cut-off value	Sensitivity	Specificity
**All TE events**	0.67	0.017	8176 ng/mL	64%	71%
**VTE events**	0.71	0.011	8176 ng/mL	75%	57%

Table S4. Stratified Cox proportional-hazard model for 90-day mortality in any TE.

	HR	95% CI	*p*
**D-Dimer >8176 ng/mL**	3.03	1.26-7.26	**0.013**
Gender	3.28	1.07-10.03	0.038
Age	1.04	1.00-1.09	0.051
Alcohol consumption	1.89	0.54-6.67	0.322

CI, confidence interval; HR, hazard ratio.

Table S5. Stratified Cox proportional-hazard model for 90-day mortality in VTE.

	HR	95% CI	*p*
**D-dimer >8176 ng/mL**	3.83	1.22-12.08	**0.022**
Gender	3.55	0.95-13.26	0.059
Age	1.03	0.98-1.09	0.206
Alcohol consumption	1.92	0.51-7.16	0.022

CI, confidence interval; HR, hazard ratio.

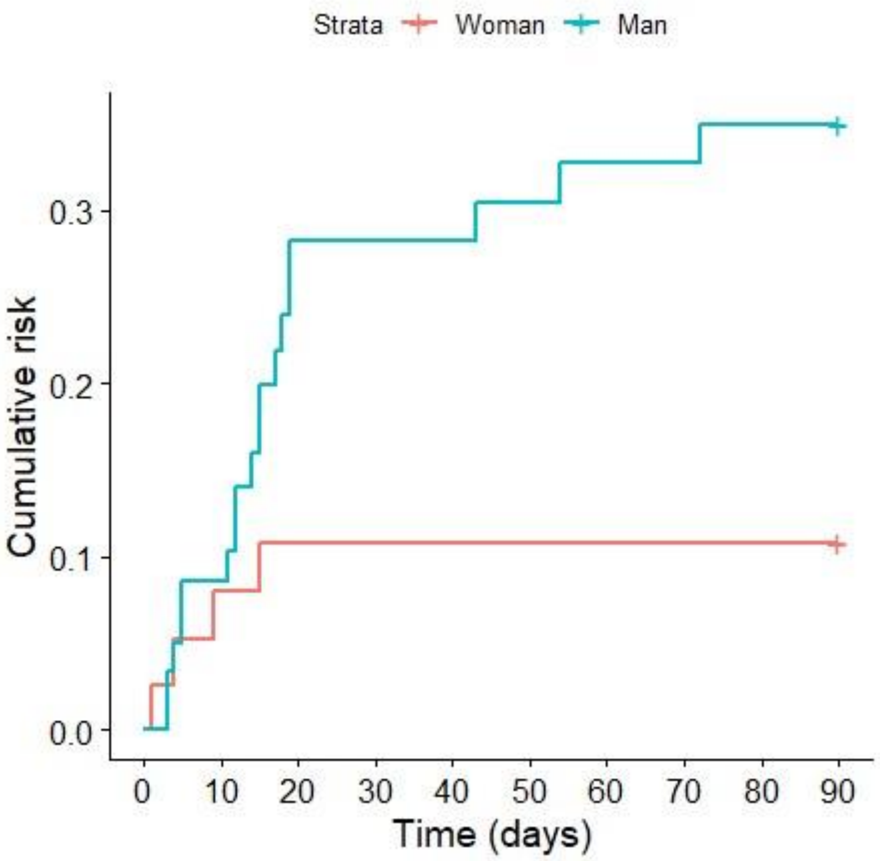

Figure S1. Cumulative risk for TEs depending on gender.

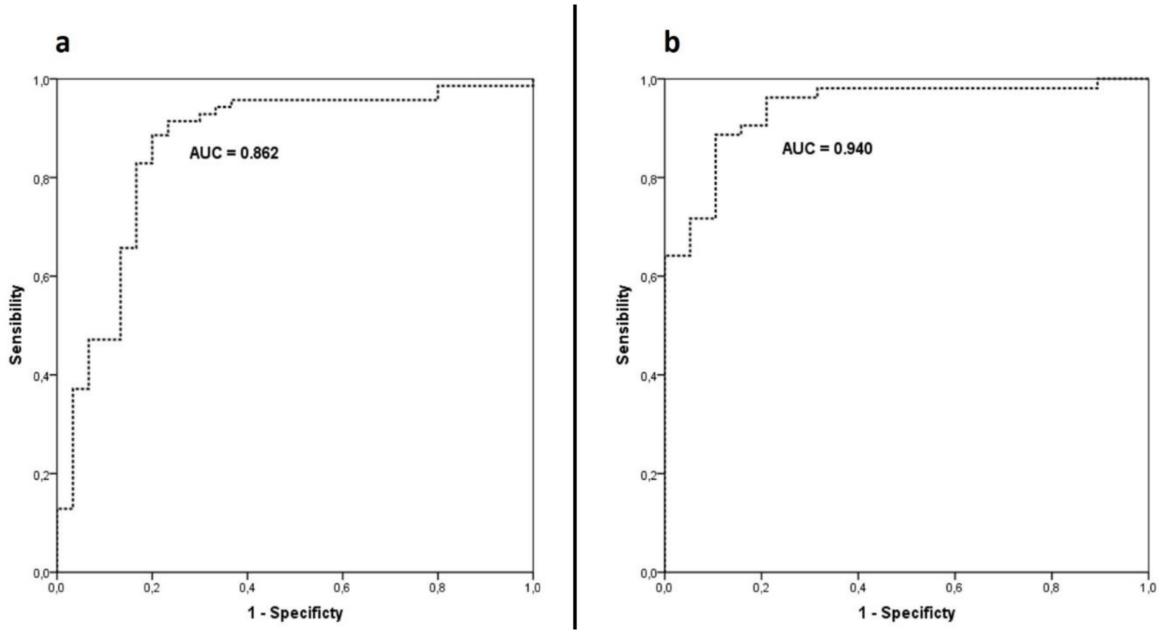

Figure S2. (a) ROC curve analysis for evaluating D-dimer accuracy to detect TEs in COVID-19 at admission. (b) ROC curve analysis for evaluating D-dimer accuracy to detect VTEs in COVID-19 at admission.

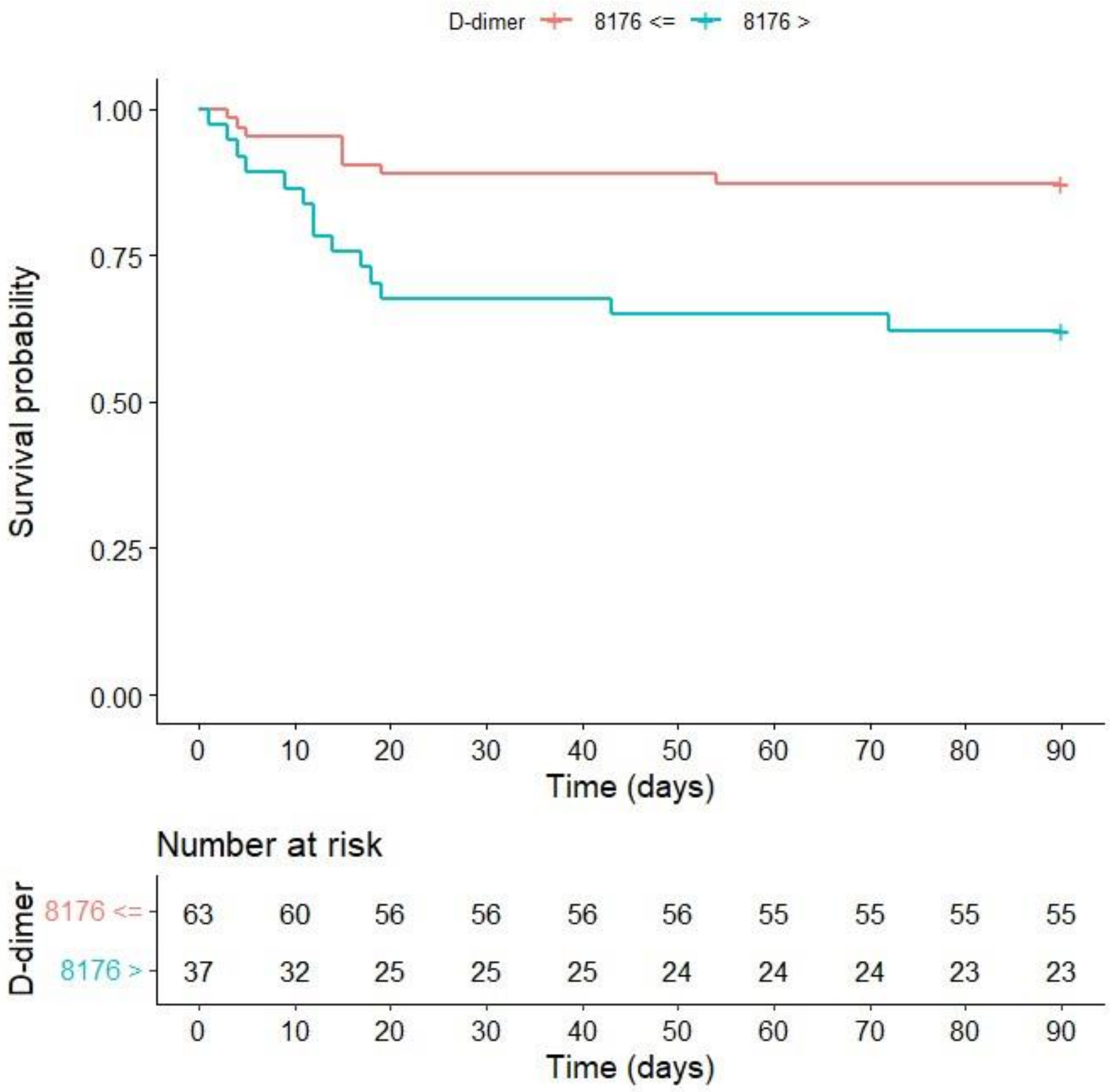

Figure S3. Kaplan–Meier survival curves in TEs for 90-day mortality.

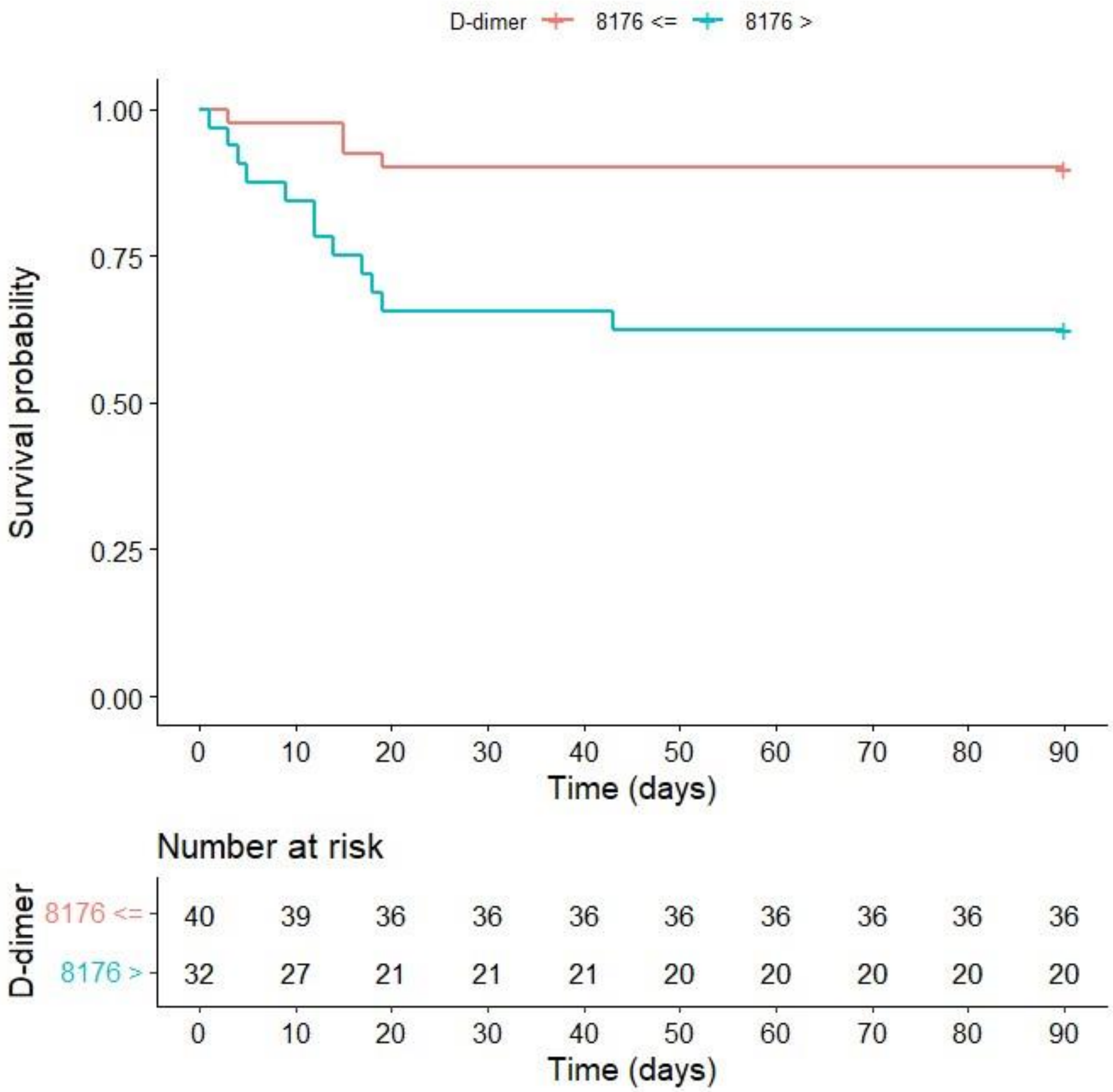

Figure S4. Kaplan–Meier survival curves in VTEs for 90-day mortality.
